# Proteomic profiling of postmortem prefrontal cortex tissue of suicide completers

**DOI:** 10.1038/s41398-022-01896-z

**Published:** 2022-04-05

**Authors:** Min Ji Kim, Misol Do, Dohyun Han, Minsoo Son, Dongyoon Shin, Injoon Yeo, Young Hyun Yun, Seong Ho Yoo, Hyung Jin Choi, Daun Shin, Sang Jin Rhee, Yong Min Ahn, Youngsoo Kim

**Affiliations:** 1 Department of Neuropsychiatry, Seoul National University Hospital, 101 Daehak-ro, Seoul, Korea; 2 Department of Psychiatry and Behavioral Science, Seoul National University College of Medicine, 101 Daehak-ro, Seoul, Korea; 3 Department of Biomedical Engineering, Seoul National University College of Medicine, 103 Daehak-ro, Seoul, Korea; 4 Biomedical Research Institute, Seoul National University Hospital, 101 Daehak-ro, Seoul, Korea; 5 Department of Biomedical Sciences, Seoul National University College of Medicine, 103 Daehak-ro, Seoul, Korea; 6 Department of Anatomy and Cell Biology, Seoul National University College of Medicine, 103 Daehak-ro, Seoul, Korea; 7 Department of Forensic Medicine, Seoul National University College of Medicine, 103 Daehak-ro, Seoul, Korea

**Keywords:** Predictive markers, Medical genetics

## Abstract

Suicide is a leading cause of death worldwide, presenting a serious public health problem. We aimed to investigate the biological basis of suicide completion using proteomics on postmortem brain tissue. Thirty-six postmortem brain samples (23 suicide completers and 13 controls) were collected. We evaluated the proteomic profile in the prefrontal cortex (Broadmann area 9, 10) using tandem mass tag-based quantification with liquid chromatography–tandem mass spectrometry. Bioinformatics tools were used to elucidate the biological mechanisms related to suicide. Subgroup analysis was conducted to identify common differentially expressed proteins among clinically different groups. Of 9801 proteins identified, 295 were differentially expressed between groups. Suicide completion samples were mostly enriched in the endocannabinoid and apoptotic pathways (CAPNS1, CSNK2B, PTP4A2). Among the differentially expressed proteins, GSTT1 was identified as a potential biomarker among suicide completers with psychiatric disorders. Our findings suggest that the previously under-recognized endocannabinoid system and apoptotic processes are highly involved in suicide.

## Introduction

Suicide is a leading cause of death worldwide. According to the World Health Organization, >800,000 people die by suicide every year [1]. Among the several proposed clinical and environmental risk factors [2, 3], a biological basis for suicide seems logical considering the familial clustering of suicidal behavior, and its high heritability of 30–50% [4–7]. Previous studies have shown that suicidal states are associated with various biochemical changes affecting neurotransmitter systems (i.e., serotonin, glutamate, γ-aminobutyric acid, and their signaling pathways) [8–11], the inflammatory response [12], hypothalamic–pituitary–adrenal axis [13], and glial cell function/neurotrophic factors [14, 15]. However, considering the complex pathways leading to suicide, further research is needed to determine the biological mechanisms underlying this phenomenon.

One of the biggest obstacles to identifying the biological basis of suicidal behavior is that most previous studies were conducted in living high-risk suicide patients (i.e., suicide attempters or patients with mood disorders), whose characteristics may differ from those of suicide completers [16, 17]. Compared to suicide attempts, suicide completion is difficult to study because of its rarity, accessibility to the brain of suicide completers, and origin of the psychopathology. Several postmortem studies have aimed to provide more direct insights into the biological mechanisms underlying suicide [18], however, since death itself causes biological changes in the tissue, controlling for tissue freshness is an obstacle. In addition, confounders such as psychiatric disorders and psychotropic medications made it difficult to interpret the results in postmortem studies. The information collected from the deceased was often incomplete, leading to a low statistical power that hindered meaningful analyses.

Among postmortem studies of suicide completers, candidate gene approaches predominated so that only important proteins in major pathways were indicated as biomarkers for suicide (i.e. brain-derived neurotrophic factor or receptor tyrosine kinase B [19]). However, since candidate gene approaches may have limited scope and clinical value in a complex phenomenon like suicide, genome wide studies would be preferred [20, 21]. Proteomic profiling studies of postmortem brains from suicide completers are scarce having a small sample size of 5–20 brains and using methodologies such as transcriptomic analysis with DNA microarray or RNA-seq [22–25]. However, considering factors such as different half-lives and that post-transcriptional machineries are affected by environmental factors, proteomic methods are better suited to quantify genetic functional expression and measure disease-associated dynamic pathway changes, making them ideal for studying suicide [22, 26]. Proteomic profiling can provide quantitative information on the profiled proteins and disease mechanisms by biological pathway analysis on significant proteins. With proper bioinformatics tools, it is possible to extract functional and biological information from identified proteins and acquire biological insights into the underlying molecular mechanisms [27, 28]. Among other proteomic methodologies, mass spectrometry (MS)-based proteomic approaches are the most suitable for identifying biomarkers related to a given disease entity, based on their ability to simultaneously screen thousands of proteins to obtain hundreds of differentially expressed proteins (DEPs) in small samples [29, 30].

The current study aimed to identify alterations in the proteomic profile of the prefrontal cortex of the postmortem brain of subjects with suicidal behavior [31] using an MS-based proteomic approach. By using multiple comprehensive bioinformatics analyses, unknown biological pathways and possible biomarkers related to suicide were identified, which may help elucidate the biological basis of suicide and related psychopathologies.

## Methods

### Subjects and brain samples

Postmortem brain samples were obtained from the Seoul National University Department of Forensic Medicine. Suicide completers were defined as individuals whose cause of death was a self-inflicted injury as confirmed by a forensic expert during autopsy. The control group comprised brain samples obtained from victims of sudden death, whose cause of death was mostly cardiac, as confirmed by a forensic expert. The postmortem interval was calculated based on the estimated time of death and time of autopsy. A total of 36 brain samples were collected: 23 suicide completers and 13 sudden death controls. Fresh brain tissue samples from the dorsolateral prefrontal cortex (DLPFC, Brodmann area 9, 10) were dissected and stored at 80 °C until further use. The DLPFC was chosen considering both proven morphological differences of the part between suicide completers and controls (i.e. decreased brain volume, neuron density, and neurotransmitters [32–34]) and dysfunction of the part in people with suicide behaviors [31, 35]

Clinical information, including diagnostic estimation, was obtained from anonymized records, which contained the declaration of family, close friends, and police. Toxicological data were used to confirm this declaration. Toxicological screening, including medications and alcohol concentration, was performed using peripheral blood obtained at the time of autopsy. All information was primarily evaluated by forensic experts who participated in autopsies, as well as a psychiatrist, and the diagnosis was reviewed and confirmed by an independent experienced psychiatrist. Only samples with consensus diagnosis based on the Diagnostic and Statistical Manual of Mental Disorders, Fifth Edition (DSM-5) criteria were included.

This study protocol was approved by the Seoul National University Hospital Institutional Review Board (IRB No. 2004-216-1119)

### Measurement of tissue pH

pH is one of the most widely used parameters in tissue quality measurements [36]. Each brain tissue sample (100 mg) was homogenized and sonicated for 30 s (Sonics & Materials, Newtown, CT, USA) in 1 mL of distilled water at 4 °C. The homogenate was briefly chilled on ice, and the pH was measured with a pH meter (Thermo Scientific, Rockford, IL, USA) calibrated at 4 °C with three standards (pH 4, 7, and 10). All pH measurements were performed in duplicate.

### Tissue preparation for proteomic analysis

A total of 36 postmortem brain tissue samples were resected and subjected to a previously described sample preparation method, with some modifications [30, 37–39]. Tissue samples were washed six times with sterilized phosphate-buffered saline for 10 min each. Washed tissues were homogenized and sonicated for 30 s (Sonics & Materials, Newtown, CT, USA) in 200 µL lysis buffer (4% SDS, 2 mM TCEP in 0.1 M TEAB pH 8.5). After the samples were incubated for 20 min at 100 °C, they were centrifuged to separate the supernatant from debris and other solid contents, and the pellet was discarded. The supernatant was transferred to a 0.22-µm pore costar filter (Corning Inc., New York, NY, USA) and centrifuged. Protein concentration was measured using a bicinchoninic acid reducing agent compatibility assay kit (Thermo Scientific, Rockford, IL, USA). A pooled protein sample was prepared by mixing 34 µg aliquots from all samples.

Protein digestion was performed using a combined approach including acetone precipitation and the filter-aided sample preparation procedure [30, 37, 38]. Prior to the digestion step, the pooled protein sample and 300 µg of each tissue sample were precipitated with cold acetone (Sigma-Aldrich, St. Louis, MO, USA) at a 1:5 (sample:acetone, v/v) ratio. The mixture was incubated overnight at −20 °C after thorough vortexing. The precipitate was centrifuged for 10 min at 15,000 rpm at 4 °C, and acetone was gently removed. After an additional rinse step with 500 µL of cold acetone and centrifugation, the protein pellet was air-dried for 2 h and stored for digestion. Acetone-precipitated samples (one pooled tissue sample and 36 individual tissue samples) were mixed with 30 µL denaturation buffer (4% SDS, 0.1 M DTT, 0.1 M TEAB, pH 8.5). After gentle vortexing, the mixture was boiled for 30 min at 95 °C for protein denaturalization. The denatured proteins were mixed with 300 µL 0.22-µm-pore filtered UA buffer (8 M urea, 0.1 M TEAB, pH 8.5) and then transferred to a 30-kDa centrifugal filter (Millipore, Billerica, MA, USA). The sample was then centrifuged three times (14,000×*g*, 15 min, 20 °C) to remove SDS. The washed samples were incubated in 200 µL of 50 mM iodoacetamide in UA buffer at room temperature for 30 min to alkylate the reduced cysteine. After the UA buffer was exchanged with 40 mM TEAB, samples were digested with 0.1 µg/µL trypsin/Lys-C at a ratio of 1:100 (enzyme:substrate, wt/wt) for 18 h at 37 °C. To estimate the amount of peptides, tissue digestions were measured by a tryptophan fluorescence assay at 350 nm using an excitation wavelength of 295 nm [40].

### Tandem mass tag labeling

**Fig. 1:**
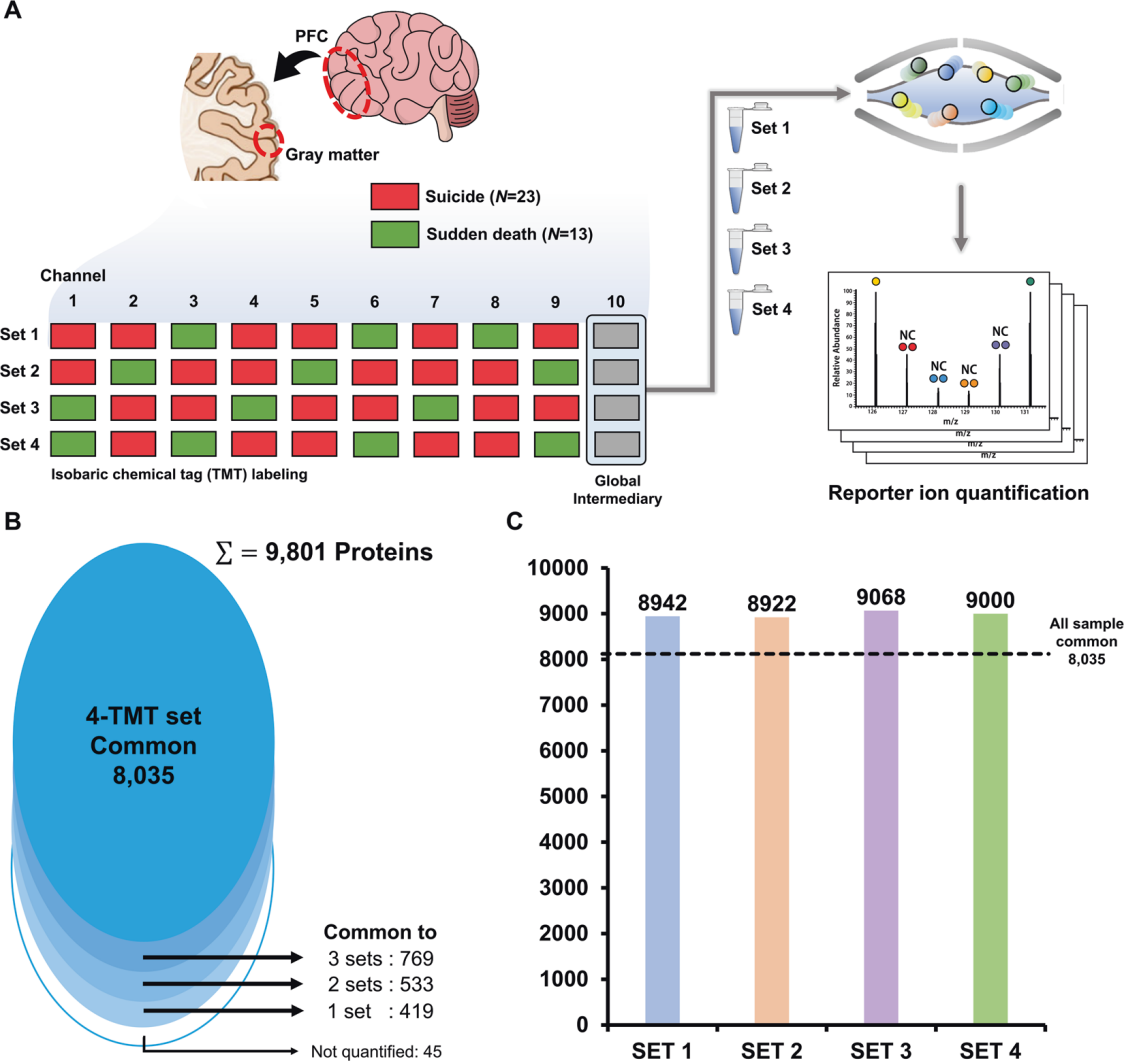
Detailed experimental workflow. **A** Distribution of the 36 samples to four tandem mass tag (TMT) experimental sets. Each experimental set comprised nine samples and one common pooled sample. **B** A total of 8035 proteins were quantified in all four experimental sets. **C** About 9000 proteins were identified similarly in all four experimental sets.

Because of the limited number of tandem mass tag (TMT) channels, we decided to distribute the 36 samples in four TMT experimental sets. After all samples were randomly distributed, each experimental set had nine individual samples and one pooled sample (Fig. 1A). Prior to the labeling step, 40 mM TEAB buffer was added to each 30-µg peptide sample to equalize the volume. Then, the same ovalbumin volume was added to the samples. TMT reagents (0.8 mg) were dissolved in 110 µL of anhydrous ACN, of which 25 µL was added to identical channels in the four experimental sets. Then, 38 µL of ACN was added to achieve a final concentration of 30%. The mixture was incubated for 75 min at 23 °C. The reaction was quenched with hydroxylamine to a final concentration of 0.3% (v/v) for 15 min at 23 °C. TMT-labeled samples in one set were pooled and lyophilized to dryness in a speed-vacuum centrifuge.

### Sample desalting and high-pH revered-phase peptide fractionation

TMT-labeled samples were re-dissolved in 0.1% trifluoroacetic acid and desalted using an HLB OASIS column according to the manufacturer’s instructions. Consequently, high-pH reversed-phase peptide fractionation was performed using an Agilent 1260 bioinert HPLC (Agilent, Santa Clara, CA, USA) equipped with a 300 Extended-C18 column (4.6 mm I.D. × 25 cm long, 5 µm C18 particle). Peptide samples were separated with a 60 min linear gradient from 5% to 40% ACN in 15 mM ammonium hydroxide at a flow rate of 0.4 mL/min. The sample was fractionated into 96 fractions, concatenated into 24 fractions with varying hydrophobicities to optimize coverage and liquid chromatography–tandem mass spectrometry (LC–MS/MS) run time. The fractions were lyophilized in a vacuum centrifuge and stored at −80 °C until LC–MS/MS analysis.

### RP-nano LC–ESI–MS/MS analysis

Peptide samples were analyzed using a Q-Exactive mass spectrometer equipped with an EASY-Spray ion source (Thermo Fisher Scientific, Waltham, MA, USA) coupled with an Easy-nano LC 1000 (Thermo Fisher Scientific, Waltham, MA, USA), following our established protocol [30, 37, 38]. The peptides were separated on a 2-column setup that comprised a trap column (Thermo Fisher Scientific, 75 µm I.D. × 2 cm long, 3 µm Acclaim PepMap100 C18 beads, 100 Å) and an analytical column (Easy-Spray PepMap RSLC, 75 µm I.D. × 50 cm long, 3 µm ReproSil-Pur-AQ C18 beads, 100 Å). Prior to sample injection, the dried peptides were re-dissolved in solvent A (2% ACN and 0.1% formic acid). The peptide samples were separated with an ACN gradient (6–90%) run for 210 min by mixing solvent A (2% ACN and 0.1% v/v formic acid) and solvent B (100% acetonitrile and 0.1% v/v formic acid) in varying proportions in all samples. Peptides eluted from the analytical column were ionized at a spray voltage of 2.0 kV in positive ion mode. Mass spectra were collected in a data-dependent acquisition mode using the top 15 method with 70,000 resolution at *m*/*z* 200 with a mass range of 350–1700*m*/*z*. The automatic gain control (AGC) target value was 3 × 10^6^ and the isolation window for MS/MS was 1.2*m*/*z*. The 15 most abundant ions were fragmented by higher-energy collisional dissociation with a normalized collision energy of 33 at a resolution of 35,000 at *m*/*z* 200. The AGC target value for MS/MS was 5 × 10^5^. The maximum ion injection times for the survey and MS/MS scans were 30 and 120 ms, respectively. Dynamic exclusion was set at 40 s to prevent repeated sequencing.

### Computational MS data analysis

The raw MS files were processed using Proteome Discoverer version 2.2 (Thermo Fisher Scientific, Waltham, MA). The tandem mass spectra search was conducted using the SEQUEST-HT algorithm against the UniProt *Homo sapiens* database (December 2014 released 88,717 protein entries, http://www.uniprot.org), which included an additional protein, chicken ovalbumin. The database search was conducted with the following parameters: full enzyme digestion using trypsin (after KR/−) up to two missed cleavages; a precursor ion mass tolerance of 20 ppm (monoisotopic mass); a fragment ion mass tolerance of 0.02 Da (monoisotopic mass); static modifications of 229.163 Da on lysine residues and peptide N-termini for TMT labeling and 57.02 Da on cysteine residues for carbamidomethylation; and dynamic modifications of 42.01 Da for protein N-term acetylation and 15.99 Da for methionine oxidation. In accordance with the established target-decoy search strategy [41], the search results were filtered at a false discovery rate of <1% to identify peptides and proteins. Proteins were quantified by calculating reporter ion relative intensities using the “Reporter Ions Quantifier” node in Proteome Discoverer. The co-isolation threshold was set to 70%. All generated proteomic data were deposited in the ProteomeXchange Consortium (http://proteomecentral.proteomexchange.org) via the PRIDE partner repository, under identifier PXD029845 [42, 43].

### Statistical analysis

The clinical variables of suicide completers and sudden death individuals were compared using the Mann–Whitney *U* test for continuous variables and Pearson’s chi-square or Fisher’s exact test for categorical variables. Statistical analyses of proteomic data were performed based on normalized protein abundance using Perseus [44]. Initially, the total identified proteins were filtered based on 8035 proteins quantified in all samples. Student’s *t*-test (*P* < 0.05, fold change [FC] > 1.2) was applied to identify significantly altered proteins. Normalized protein abundance was subjected to *z*-normalization followed by hierarchical clustering.

To confirm whether the identified DEP was due to suicide itself or other related clinical factors, sub analyses were performed among suicide completers. Suicide completers were divided into groups according to history of psychiatric disorder, history of treatment with psychotropic medications, and alcohol intake. DEPs with *p* < 0.05, and FC > 1.2 were identified among subgroups of suicide attempters.

### Bioinformatics analysis

Protein Gene Ontology (GO) was classified using the DAVID bioinformatics tool (version 6.8) [45]. The GO classification was evaluated by Fisher’s exact test to obtain a set of *p*-values, which were then filtered at a cutoff value of 0.05. Ingenuity pathway analysis (IPA) was used for functional analysis (Ingenuity Systems, http://www.ingenuity.com/). Fisher’s exact test (*P* < 0.05) was used in IPA to estimate the probability that a specific set of proteins was related to a certain pathway. The proteins included in each module were subjected to network analysis using IPA. Gene set enrichment analysis (GSEA) was performed using the GSEA software (GSEA version 4.1.0). The c5.go.bp.v7.4.symbols.gmt gene set was selected as the reference gene set. Weighted gene co-expression network analysis (WGCNA), a systems biology method to understand correlation patterns among genes across samples, was adopted to identify co-expressed proteins and modules associated with suicide and sudden death (Version 1.69 in R). The eigengene was then used to summarize modules, and the association between these modules and sample traits, such as clinical parameters, was investigated [46].

## Results

### Subjects and brain samples

In total, 36 postmortem brain samples were collected, including 23 suicide completers and 13 sudden death individuals. Demographic and clinical information are presented in Table 1. There was no significant difference between suicide completers and sudden deaths in terms of age (*p* = 0.580). However, the sex ratio among suicide completers was nearly 1:1, while only men died of sudden death. The suicide method varied among suicide completers: 16 (69.6%) by drowning, 2 (8.7%) by jumping, 1 (4.3%) by crashing, and 4 (17.4%) by hanging. Among suicide completers, 12 (47.8%) had psychiatric disorders: 7 (30.4%) had schizophrenia, and 4 (17.4%) had major depressive disorder. The toxicological results showed that 13 (56.5%) subjects had not been taking psychotropic drugs, six were taking antipsychotics, and two were on antidepressants. Among suicide completers, 6 (26.1%) subjects drank alcohol at the time of suicide. The mean blood alcohol concentration of suicide victims was 0.092 (SD = 0.023), which did not differ from that of people who died of sudden death. There was no significant difference between groups in postmortem interval and measured pH (Fig. S1 and Table S1).

**Table 1:** Clinical characteristics of suicide completers and sudden death group.

Characteristic	Suicide (*N* = 23)	Sudden death (*N* = 13)	*P*
Age, mean (SD)	48.8 (13.6)	51.5 (12.9)	0.580^a^
Sex, no. (%)			0.001
Male	11 (47.8)	13 (100)	
Female	12 (52.2)	0 (0)	
Type of suicide, no. (%)			
Drowning	16 (69.6)	–	–
Jumping	2 (8.7)	–	–
Crashing	1 (4.3)	–	–
Hanging	4 (17.4)	–	–
Psychiatric disorder, no. (%)			0.112
Schizophrenia	7 (30.4)	2 (15.4)	
Major depressive disorder	4 (17.4)	0 (0)	
None	12 (52.2)	11 (84.6)	
Psychotropic medications, no. (%)			0.264
Antipsychotics	6 (26.1)	1 (7.7)	
Antidepressants	2 (8.7)	0 (0)	
Analgesics	1 (4.3)	0 (0)	
Hypnotics	1 (4.3)	0 (0)	
None	13 (56.5)	12 (92.3)	
Alcohol intake at death, no. (%)			0.439
Yes	6 (26.1)	5 (38.5)	
No	17 (73.9)	8 (61.5)	
Alcohol concentration at death (mg/dl), mean(SD)	0.092 (0.023)	0.085 (0.049)	0.931^a^
Postmortem interval, mean(SD)	52.0 (22.9)	45.8 (19.4)	0.711^a^
pH, mean (SD)	6.88 (0.19)	6.75 (0.38)	0.239^a^

*SD* standard deviation.

^a^Mann–Whitney *U* test.

### In-depth quantitative proteomics of postmortem brain tissue

We performed a quantitative proteomic analysis on 36 postmortem brain tissue samples. Because the number of samples exceeded that of the TMT channels, we decided to distribute the 36 samples into four TMT experimental sets. After all samples were randomly distributed, each experimental set had nine individual samples and one pooled sample (Fig. 1A). Briefly, each brain tissue sample was homogenized and digested, and the resulting peptides were labeled with TMT reagents. TMT-labeled samples were analyzed using a Q-Exactive mass spectrometer. A total of 96 raw files (24 peptide fractions × 4 TMT-mix experimental sets) were processed in Proteome Discoverer based on the SEQUEST-HT algorithm. A total of 9801 protein groups were identified; among them, 8035 protein groups were successfully identified in all 36 samples (Fig. 1B and C, Table S2). Our proteomic platform allowed us to discover an in-depth proteome with a dynamic range covering >6 orders of magnitude (Fig. S2). Box plot analysis was used to compare the log2-transformed intensities of all TMT channels. The median log2-transformed intensity was almost identical across the 40 TMT channels, which indicates that the quantitative analysis had no biases toward different samples (Fig. S3A). Principal component analysis of intensity values was conducted on 8,035 quantitative proteins to assess the bias according to each TMT set (Fig. S3B). The first two principal components explained 11.1% and 26.9% of the variance within the dataset, respectively. No TMT channel was clustered in accordance to TMT sets but randomly distributed. To evaluate the reproducibility of the pooled samples, we calculated the Pearson correlation coefficients of intensity values between the four pooled samples (Fig. S3C). The Pearson correlation coefficient (>0.990) between pooled samples indicated high reproducibility of the quantitative analysis.

### Differentially expressed proteins between suicide completers and sudden death individuals

**Fig. 2:**
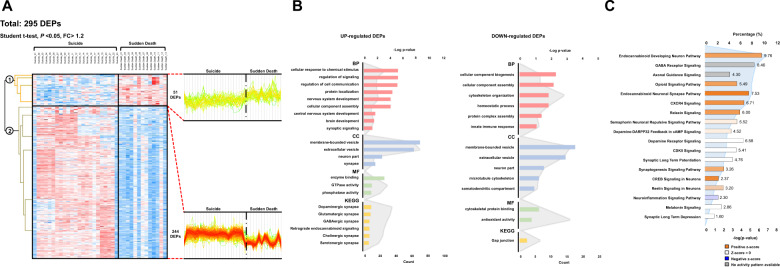
Differentially expressed proteins and GO, IPA analysis results. **A** Hierarchical heat map clusters of significant proteins by Student’s *t*-test **B** GO analysis using 244 upregulated DEPs and 51 downregulated DEPs. Each colored bar graph indicates the enriched terms in biological process (BP), cellular component (CC), molecular function (MF), and KEGG pathway. The number of participating proteins is shown on the lower axis, and log *p*-value for the upper axis (gray line graph) for GO terms. **C** Ingenuity pathway analysis (IPA) results for functional analysis. The percentage of participating proteins is shown on the upper axis, and the log *p*-value for canonical pathways in the lower axis (blue line graph). The *z*-score of each canonical pathway is represented by bar colors.

Of the 9801 identified proteins, 8035 were quantifiable in individual brain tissue samples and were used for statistical analysis. To identify DEPs, Student’s *t*-test (*P* < 0.05) was performed between suicide completers and sudden death individuals. The results, including statistical significance, *p*-values, and fold-changes, are presented in Table S3. A total of 295 DEPs were identified. Among them, 244 proteins were upregulated and 51 downregulated in suicide completers compared to sudden death individuals (Fig. 2A). The variance in expression between suicide completers and sudden death individuals is depicted in a volcano plot (Fig. S4).

According to the GO-enrichment analysis among 244 upregulated DEPs (Fig. 2B), regulation of signaling, regulation of cell communication, protein localization, and nervous system development were the most significantly enriched biological processes; membrane-bound and extracellular vesicles, neurons, and synapses were the most significantly enriched cellular components; and enzyme binding, GTPase, and phosphatase activity were the most significantly enriched molecular functions. In KEGG enrichment analysis, almost all kinds of known neurotransmitter synapses were obtained: dopaminergic, glutamatergic, GABAergic, cholinergic, serotonergic, and endocannabinoid.

GO-enrichment analysis of the 51 downregulated DEPs resulted in similar pathways (Fig. 2B). Cellular component biogenesis and assembly, cytoskeleton organization, and protein complex assembly were significantly enriched biological processes; membrane-bound vesicles, extracellular vesicles, neuron parts, microtubule cytoskeleton, and somatodendritic compartment were the most enriched cellular components; and cytoskeletal protein binding and antioxidant activity were the most enriched molecular functions. A gap junction was obtained in KEGG enrichment analysis. A complete list of related GO terms is available in Online Supplementary Table 4.

### Ingenuity pathway analysis and gene set enrichment analysis

Several highly enriched canonical pathways were identified with IPA, including the endocannabinoid developing neuron pathway, opioid signaling pathway, and endocannabinoid neuronal synapse pathway (Fig. 2C). Neurotransmitter systems, including the GABA receptor signaling pathway, dopamine receptor signaling, and dopamine–DARPP32 feedback pathway were also identified. Axonal guidance signaling, synaptic long-term potentiation, and synaptogenesis pathways were discovered along with the neurotransmitter pathways in IPA.

In addition, the differentially expressed gene sets in suicide completers compared with controls detected by GSEA, their normalized enrichment score, and nominal *p*-value are shown in Figs. S5 and S6. Regulation of synaptic vesicle exocytosis and neurotransmitter transport are the two most significant biological processes identified. Similarly, processes related to neurotransmitter uptake, response to catecholamines or dopamine, neuron death, projection, and regeneration were enriched in the DEPs discovered. In contrast, pathways associated with the positive regulation of the apoptotic signaling pathway and maintenance of protein localization were downregulated.

### Weighted gene co-expression network analysis

**Fig. 3:**
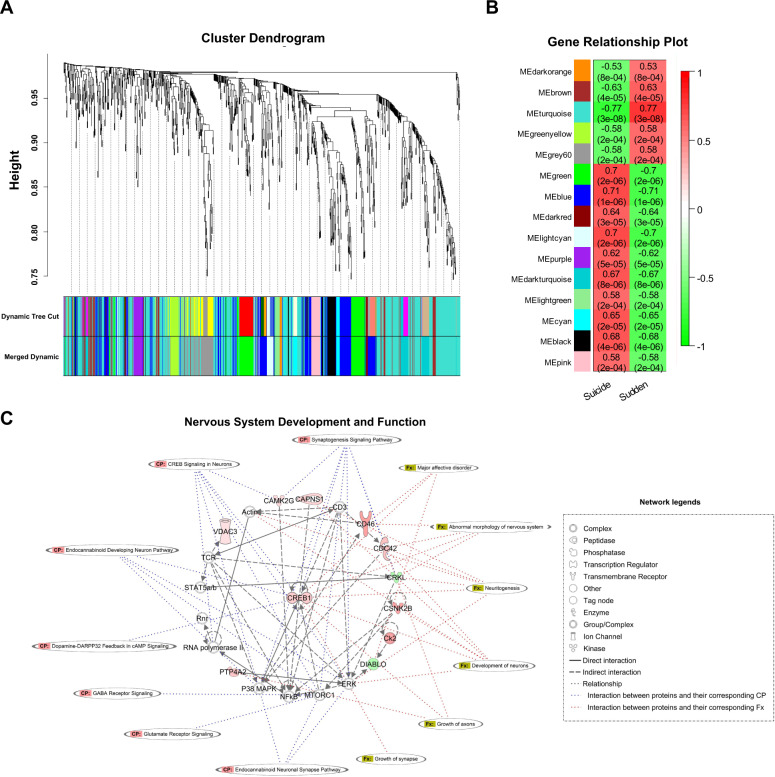
WGCNA analysis. **A** Optimal cluster sets obtained by dynamic tree cutting and automatic cluster merging. **B** Heatmaps showing correlation of module eigengenes. Pearson correlation coefficients of each module are shown and differently colored. **C** The top network of nervous system development and function was identified using 154 proteins in the blue module, which had the largest correlation with the suicide group.

A hierarchical clustering dendrogram of all samples is depicted in Fig. S7A. We chose the soft threshold power, *β* = 12, to construct the unsigned co-expression network (*R*^2^ = 0.7) (Fig. S7B and C). A topological overlap matrix (TOM) was calculated to form the network adjacency matrix, and hierarchical clustering was performed based on the TOM distance (dissTOM = 1–TOM). Optimal cluster sets were obtained by dynamic tree cutting, and closely correlated clusters were merged using the automatic cluster merging function (Fig. 3A). Subsequently, using WGCNA analysis, 15 co-expressed modules were identified in our dataset (Fig. 3B).

The dataset had two very large modules (blue and turquoise) and several smaller modules, indicating that the expression levels of numerous genes were strongly correlated (Table S5). The blue and turquoise modules had the largest Pearson correlation coefficients in the suicide and sudden death groups, respectively. Subsequently, we examined the association between the blue module and suicide completers and between the turquoise module and sudden death individuals through IPA network analysis. The top network using 154 proteins in the blue module that had the largest correlation with the suicide group was nervous system development and function; each protein in the network had a good correlation between protein and trait (protein significance, PS), and module (module membership, MM) (Figs. 3C and S8). The proteins included in the nervous system development and function networks are associated with canonical pathways, neurotransmitter signaling, and neuron development. Proteins with highest significance for suicide were CAPNS1, CDC42, PTP4A2, CD46, and CSNK2B, and were connected to CREB1, ERF, and NF-KB.

The top network using 393 proteins in the turquoise module having the largest correlation with the sudden death group was organismal injury and abnormalities; each protein in the network demonstrated high PS and MM values (Fig. S9). The proteins included in the organismal injury and abnormalities network were associated with canonical pathways and functions related to malfunction of various organs and apoptosis.

### Subgroup analyses according to clinical factors

In subgroup analyses, certain proteins related to clinical characteristics (i.e., psychiatric disorders, psychotropic use, and alcohol intake) were identified among suicide completers (Table 2). With respect to the DEP list obtained comparing suicide and sudden death, there were some common proteins. For instance, GSTT1 was identified among suicide completers who had psychiatric disorders; ABRACL and UBE2D3 were identified among schizophrenic suicide completers; COL4A1 and CD82 were identified among people who took antipsychotics; and RPS4Y1, S100A10, and MRGPRF were identified among people who consumed alcohol at the time of death.

**Table 2:** Proteins related to clinical characteristics among suicide completers.

Comparative groups	Psychiatric disorders			Medications		Alcohol intake
	Psychiatric disorder vs. No disorder (11: 12)	Schizophrenia vs. No disorder (7: 12)	MDD vs. No disorder (4: 12)	Psychotropics vs. No medication (10: 13)	Antipsychotics vs. No medication (6: 13)	Alcohol intake vs. No-alcohol (6: 17)
Number of DEPs (Student’s t-test P < 0.05 & FC > 1.2)	4	13	8	5	12	34
Gene names	NIPSNAP3B	EPB41L1	FGG	BCAS1	CEP170B	GFAP
	GSTT1^a^	NIPSNAP3B	NIPSNAP3B	NIPSNAP3B	EPB41L1	CACNA1E
	TSPAN8	CHI3L1	ACBD3	NQO1	EPB41L1	SLC1A3
	NQO1	STBD1	DCHS1	GPNMB	BCAS1	COL6A1
		EMILIN2	GSTT2	APOA2	CFAP36	ANK3
		SLC14A1	LRP2		COL4A1^a^	RASA4
		MORF4L2	ABCC10		CD82^a^	COL6A3
		NELFE	CHRNA4		STBD1	RPS4Y1^a^
		TSPAN8			MORF4L2	SLC7A11
		CDKN2AIP			NELFE	CD44
		NQO1			TTR	MICALL2
		ABRACL^a^			GPNMB	FAM92A1
		UBE2D3^a^				S100A10^a^
						TSPAN15
						**MRGPRF**^**a**^
						MAN2A1
						IGHG1
						IGHG1
						SERPINA1
						IGHA1
						IGKV3-11
						IGHM
						HPX
						IGLC2
						IGLL5
						IGHM
						IGKV3-20
						AHSG
						IGKV2D-28
						IGKV1D-33
						IGKV2-30
						IGLV3-21
						SYT6
						CD5L

*DEP* differentially expressed protein, *MDD* major depressive disorder.

^a^Proteins common to suicide vs. normal analyses.

## Discussion

In the current study, we identified protein profiles related to suicide and sudden death using postmortem brain tissue. Using bioinformatics tools, we discovered canonical pathways related to suicide completion, such as various neurotransmitter transmission signaling pathways, including endocannabinoid pathways. Through subgroup analyses, we found common proteins between suicide completion and other factors related to suicide such as psychiatric disorders, psychotropic use, and alcohol intake [47, 48].

To confirm the reliability of our study results, comparative analysis with other studies was conducted. The most recent postmortem brain tissue study using a proteomic approach identified 5644 proteins using label-free proteomics acquisition through nano-flow liquid chromatography–electrospray tandem MS in five samples in the suicide and non-suicide group [22], while our study identified 8035 proteins. There were 4337 overlapping proteins between our study and Cabello-Arreola’s study. A Venn diagram for the comparison is shown in Fig. S10A. Considering the variability in experimental conditions in proteomics, it is reassuring that other studies have documented similar suicide-related proteins. In addition, since more proteins were identified in the current study, the reliability of this study increased compared to that of previous studies. When comparing the functional pathways found in two studies [22, 49], many pathways related to neurotransmission and synaptic signaling/remodeling overlapped, further increasing the validity of the current results (Fig. S10B). Among the various pathways, the endocannabinoid signaling pathway, and GABA receptor signaling pathway were the two most enriched pathways.

Among the various pathways identified, the endocannabinoid system has received increasing attention due to its role in the regulation of affective disorders. Previous studies have shown higher cannabinoid receptor type 1 (CB1) and CB1-mediated G-protein activation in the DLPFC of depressed suicidal subjects [48]. Mechanistically, CB1 and cannabinoid receptor type 2 receptors inhibit the presynaptic release of neurotransmitters, such as GABA and glutamate [50, 51], which reflects the cross-talk between neurotransmitter pathways. Using multiple bioinformatics analyses, we could identify pathways related to the endocannabinoid system. The fact that cannabidiol interacts with brain-derived neurotropic factor (BDNF), which responds to inflammation, stress, and other imbalances and rapidly mediates synaptic plasticity in the medial prefrontal cortex through the BDNF–TrkB signaling pathway [52, 53] also supports our result that endocannabinoid systems are related to neuronal development and synaptogenesis. Whether these changes are an adaptive mechanism or a pathological process remains to be clarified; however, it is intriguing to reinforce an under-recognized endocannabinoid system which was as a novel biomarker candidate for suicide. In addition, GABAergic alteration of gene expression in suicide attempters has been extensively replicated [38, 39], albeit with scarce proteomic evidences were scarce. Since epigenetic modifications areis active in suicidale brains, and the GABAergic system is susceptible to epigenetic modifications [40], the current result of proteomics would present a more stronger evidence than that of previous studies.

Interestingly, the pathways reported in the current study included cellular degeneration, apoptosis, and related cytoskeleton structural changes. In GO analysis, the DEPs in suicidal subjects related to cellular component biogenesis and assembly were downregulated. This was also replicated in the IPA analysis where we found downregulation of the positive regulation of apoptotic signaling pathway and upregulation of the positive regulation of neuron death in the suicide group. Furthermore, the most significant proteins for suicide in the WGCNA analysis included CAPNS1 (Calpain Small Subunit 1), which is suggested to play a neuroprotective role [54], and CSNK2B and PTP4A2, implicated as biomarkers for psychiatric disorders such as major depressive disorder and schizophrenia [55–57]. They are related to the NF-κB pathway, which plays a key role in apoptosis and neuroinflammation [58–61]. This is in line with previous studies that found decreased neuronal density in Brodmann area 9 [32], and decreased microglia density in suicide attempters compared to controls [62]. Further studies are needed to investigate which cell types participate in apoptosis thus structurally and spatially modifying the brain of suicide completers.

The major concern of the current study was whether the identified DEPs were solely related to suicide completion, which is a complex phenomenon, or related to other risk factors of suicide or the result of treatment. Therefore, we performed multiple subgroup analyses. First, we compared control vs. suicide without psychiatric disorder and control vs. suicide with psychiatric disorder to prove whether having a psychiatric disorder would have influenced our results (Table S6, Figs. S11A, S11B). DEPs identified in subgroups overlapped significantly with DEPs discovered in suicide vs. control, regardless of psychiatric disorders, thereby indirectly proving that our results are minimally biased by psychiatric disorders. Second, by comparing clinical characteristics among only suicide completers, we further revealed that DEPs of suicide completion were commonly involved in key regulatory pathways not only in suicide itself but also in suicide risk factors (i.e., psychiatric disorders). GSTT1 and S100A10 are two hypothetical proteins that might be meaningfully related to both suicide completion and suicidal risk factors.

GSTT1, which plays a role in coping with oxidative stress, was identified as a biomarker among suicide completers with psychiatric disorders. Since a GSTT1 polymorphism has already been noted as a key biomarker for schizophrenia, bipolar disorder, and other mood disorders [63–65], the current result seems to provide a biological explanation of the clinical overlap between suicide and psychiatric disorders. Interestingly, the accumulation of oxidative products is known to lead to neuron degeneration implying that both suicide and psychiatric disorders are related with apoptotic pathways [66], and that GSTT1 may have a key role in biological pathways in both suicide and psychiatric disorders.

S100A10 is a protein that modulates serotonergic 1B and glutamate receptor function and has been known to be related with suicide in many previous studies [67, 68]. Further, in a mouse model, alcohol intoxication induced transcriptomic change in S100A10 [69]. S100A10 may be a possible marker for suicide in people who have comorbid alcohol use disorder. Clinically, suicide attempt among people with alcohol use disorder depicts different characteristics, such as high impulsivity and violence [70]. S100A10 may act as a key biological marker to explain clinical differences between suicide and alcohol use disorder. In developing biological treatments, it will be effective to target biological pathways commonly involved in suicide itself and suicide risk factors considering their clinical overlap. In that sense, GSTT1 or S100A10 may be promising targets for future clinical studies.

There are several limitations to our study, such as the sample size and the evaluation procedure. First, the small sample size may have affected our analyses, particularly the subgroup analyses, with respect to statistical power. Moreover, the lack of standardized diagnostic evaluation after death raises a concern for suboptimal diagnosis. However, in 34.5–61.8% of suicide cases, there was no psychiatric diagnosis is East Asia, including China, Japan, and South Korea [71–73], which is significantly higher than that of the US of 10% [74], proving that our method would be reasonable. It is possible that psychiatric disorders had not been excluded in the controls, and some patients with bipolar disorder may have been mistakenly referred to as having major depressive disorder, owing to the shared psychopathology of depressive episodes. This is because the evaluation depends on limited materials such as declarations of family, friends, and police and toxicological data.

Second, there was a limitation in collecting control group samples given the male predominance for sudden death, already shown in previous epidemiologic studies [75, 76]. To confirm whether the DEPs are caused by suicide completion itself rather than the disproportionate sex differences among samples, we first compared only male suicide completers with sudden death individuals to select statistically significant DEPs. We found 282 DEPS by comparing only men in both groups (Table S6). The results were very similar to those of DEPs discovered in suicide including both sexes and sudden death; 251 proteins (77%) were common, as shown in Fig. S11C. A significant portion of up-regulated proteins and down-regulated proteins were also common in both group comparisons (Fig. S11D, E). Thus, we concluded that the difference in protein expression between suicide and sudden death was not due to sex but suicide itself. Based on the inference that sex minimally influenced our DEPs comparing control versus suicide, we used 295 DEPs resulting from the comparison between suicide completers of both sexes and sudden death individuals in bioinformatics analysis. Further investigation of sex differences in suicide should be done with female controls.

Third, there was a possibility of bias since we could use only the accessible postmortem brain gathered from autopsy. Most suicides in our samples were due to drowning; however, hanging accounts for >50% of suicides in South Korea [77]. In addition, since most suicide completer cases were due to drowning, the low temperature of the water slowed down the decaying process, which resulted in a longer postmortem interval than other previous studies using postmortem brains. However, we measured the pH for a better characterization of tissue conditions. To objectively measure postmortem intervals despite the various suicide methods, experimental methods such as metabolic profiling should be adopted in future studies [78].

Fourth, significant confounders may have influenced our results. We tried to obtain all clinical information available from the police and the family of the deceased; however, the subject’s state right before suicide was still elusive. Although we performed several subgroup analyses to determine whether the differences in protein expression were affected by the given information (i.e., psychiatric disorders, medication, or alcohol intake), other unpredictable confounders such as undiscovered comorbidity might have affected the results (i.e., binge eating disorder, substance use disorder). Furthermore, we were unable to obtain information on the chronic use of substances such as alcohol, nicotine, and illicit drugs. Considering that such information may influence the biological pathways we discovered (such as the endocannabinoid pathway) [79], they may have acted as confounders. Finally, it is difficult to conclude whether the relationship between the unveiled networks and suicide attempts was causally related.

Nevertheless, our study has several strengths. The current study was the first to apply a proteomic approach to the postmortem brains of East Asians. A total of 36 postmortem brain samples were collected. Using high-throughput TMT-based quantification with high-resolution mass spectroscopy, 9802 proteins were simultaneously identified and quantified, the highest number so far reported in the literature. Moreover, we could comprehensively reveal potential biological pathways (i.e. endocannabinoid pathways and apoptotic pathways) related to suicide completion using multiple existing bioinformatics tools.

In conclusion, our study uncovered the largest protein pool of East Asians related to suicide completion using various bioinformatics tools. Through multiple subgroup analyses, we found common biological markers between suicide completion and suicidal risk factors such as psychiatric disorders, which are one of the most important risk factors for suicide. The pathways revealed in this study are strong candidates to explain the biological basis of suicide.

## Supplementary information

supplementary figures

supplementary tables
