# Supplementary material for: Proteomic profiling of postmortem prefrontal cortex tissue of suicide completers
Source: Transl Psychiatry. 2022 Apr 5;12:142. doi: 10.1038/s41398-022-01896-z (PMC8983647; doi:10.1038/s41398-022-01896-z)
Supplement: Supplementary file 1 — supplementary figures [file 41398_2022_1896_MOESM1_ESM.docx]

**SUPPLEMENTAL DATA**

**Proteomic profiling of postmortem prefrontal cortex tissue of suicide completers**

Min Ji Kim^1, 2,#^ , Misol Do^3,#^, Dohyun Han^4^, Minsoo Son^3^, Dongyoon Shin^5^, injoon Yeo^3^, Young Hyun Yun^6^, Seong Ho Yoo^7^, Hyung Jin Choi^5,6^, Daun Shin, Sang Jin Rhee^1,2^, Yong Min Ahn^1,2,*^, Youngsoo Kim^3,5,*^

*^1^Department of Neuropsychiatry, Seoul National University Hospital, 101 Daehak-ro, Seoul, Korea*

*^2^Department of Psychiatry and Behavioral Science, Seoul National University College of Medicine, 101 Daehak-ro, Seoul, Korea*

*^3^Department of Biomedical Engineering, Seoul National University College of Medicine, 103 Daehak-ro, Seoul, Korea*

*^4^Biomedical Research Institute, Seoul National University Hospital, 101 Daehak-ro, Seoul, Korea*

*^5^Department of Biomedical Sciences, Seoul National University College of Medicine, 103 Daehak-ro, Seoul, Korea*

^6^*Department of Anatomy and Cell Biology, Seoul National University College of Medicine, 103 Daehak-ro, Seoul, Korea*

*^7^Department of Forensic Medicine, Seoul National University College of Medicine, 103 Daehak-ro, Seoul, Korea*

**SUPPLEMENTAL FIGURES**


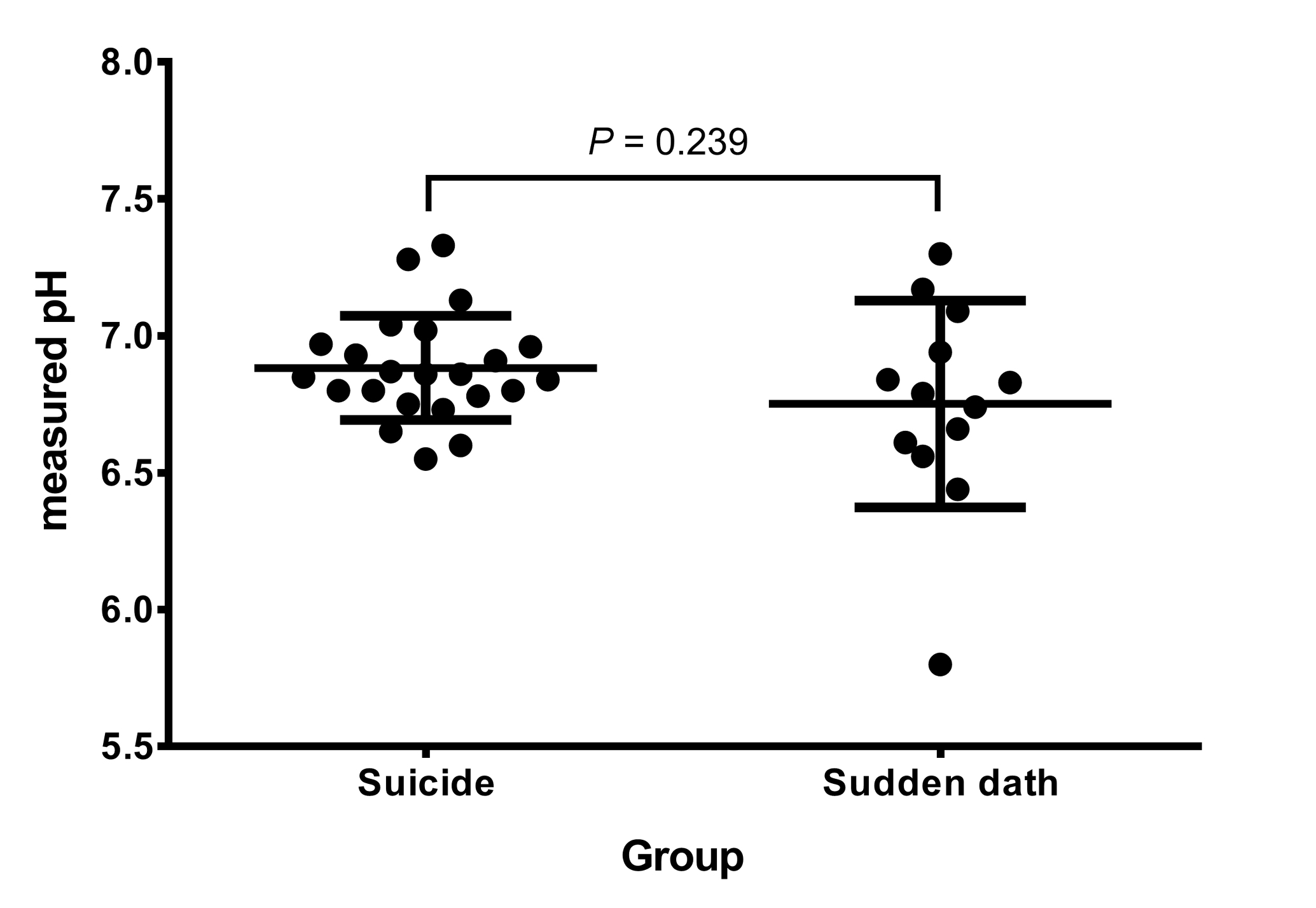


**Figure S1.** pH difference between suicide completers and sudden death group.

**
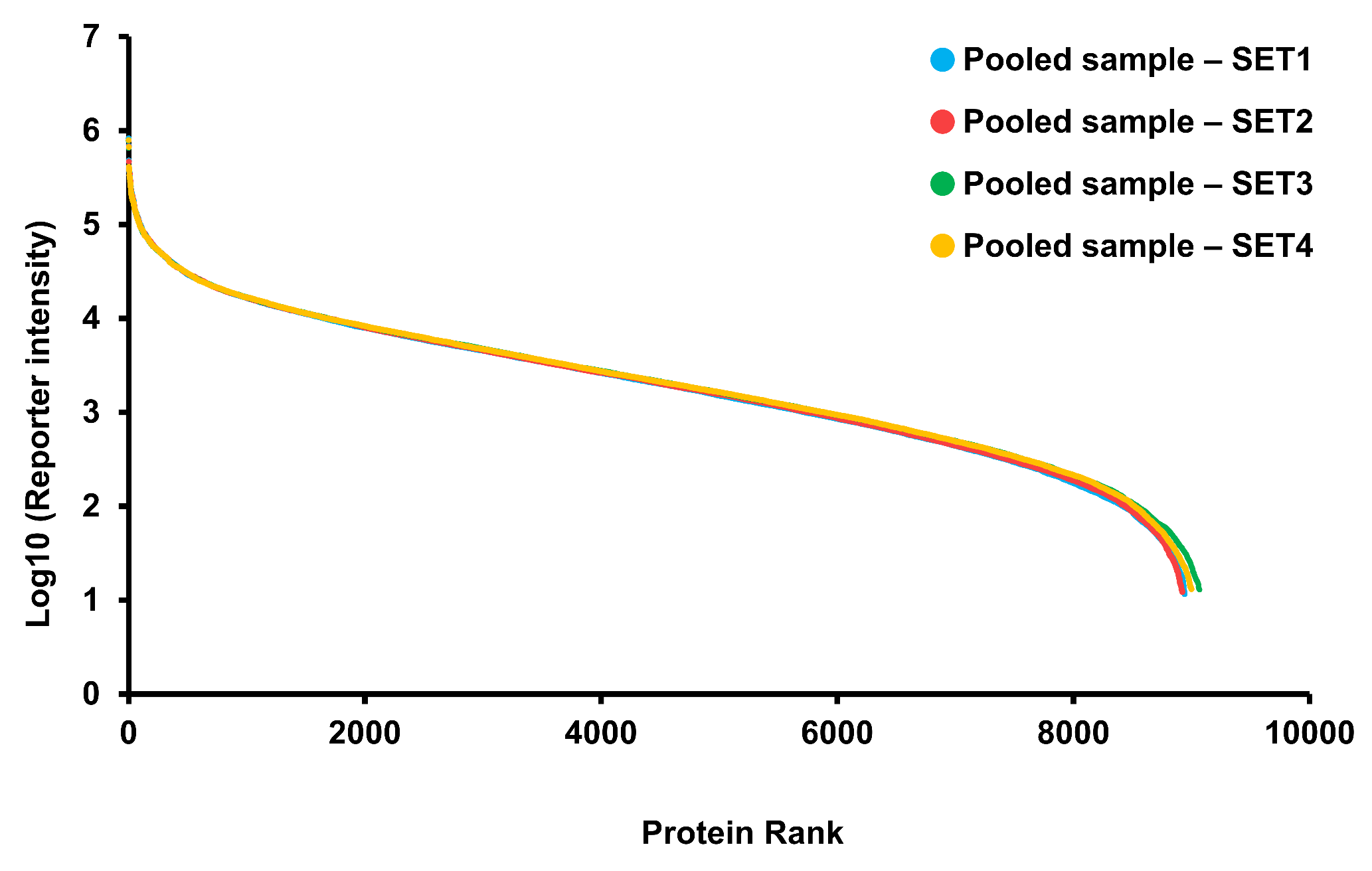
**

**Figure S2.** Dynamic range of pooled sample in four experimental sets.


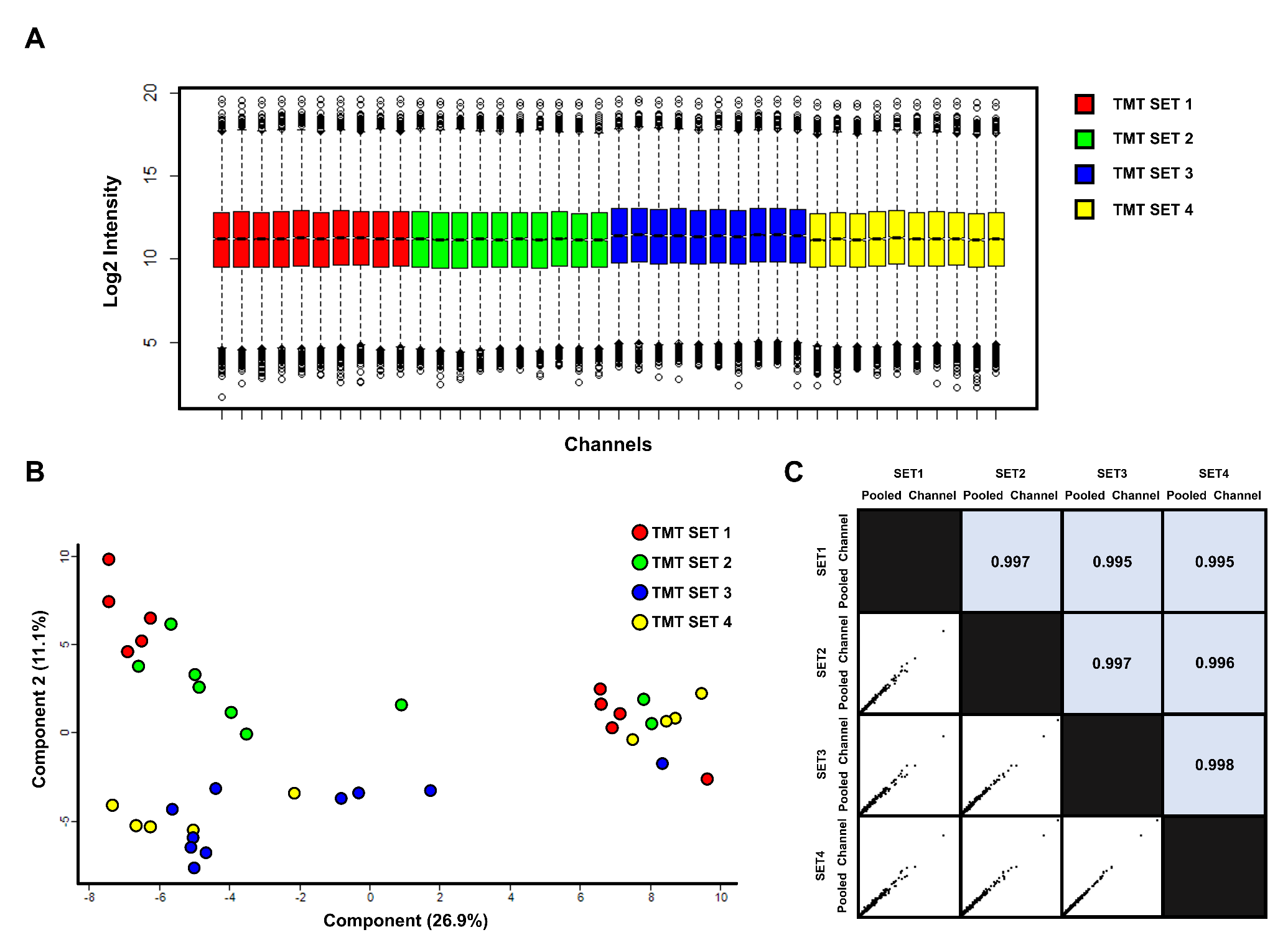


**Figure S3.** Quantitative profiling of postmortem brain tissue samples. A. Box plot of the log2-transformed intensity for each sample. B. Principal component analysis of intensity value for four experimental sets. C. Pearson correlation coefficients of intensity values between four pooled samples.


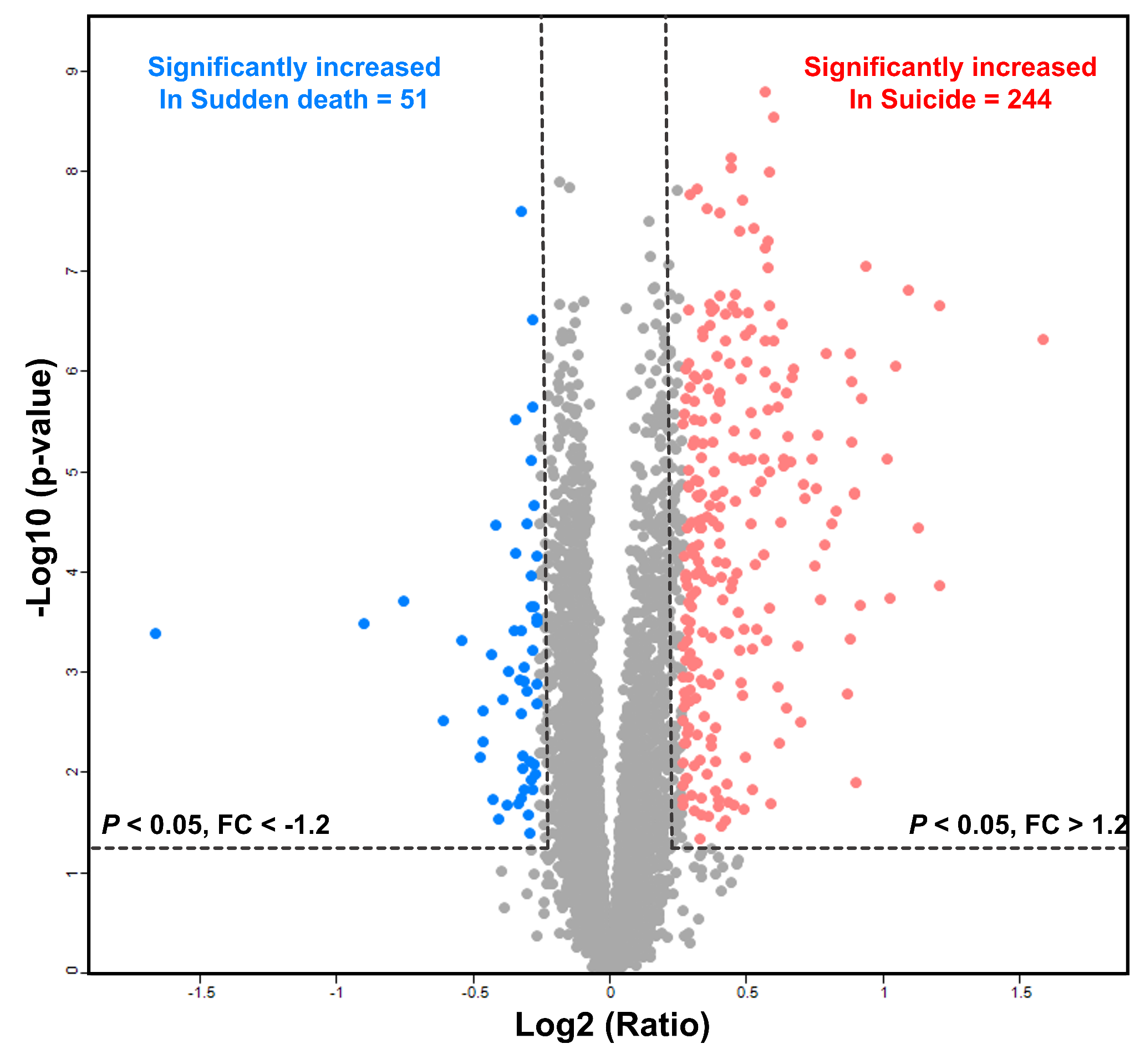


**Figure S4.** Volcano plot for differentially expressed proteins. Student’s t-test(p<0.05) was conducted for comparison between suicide completers and sudden death.


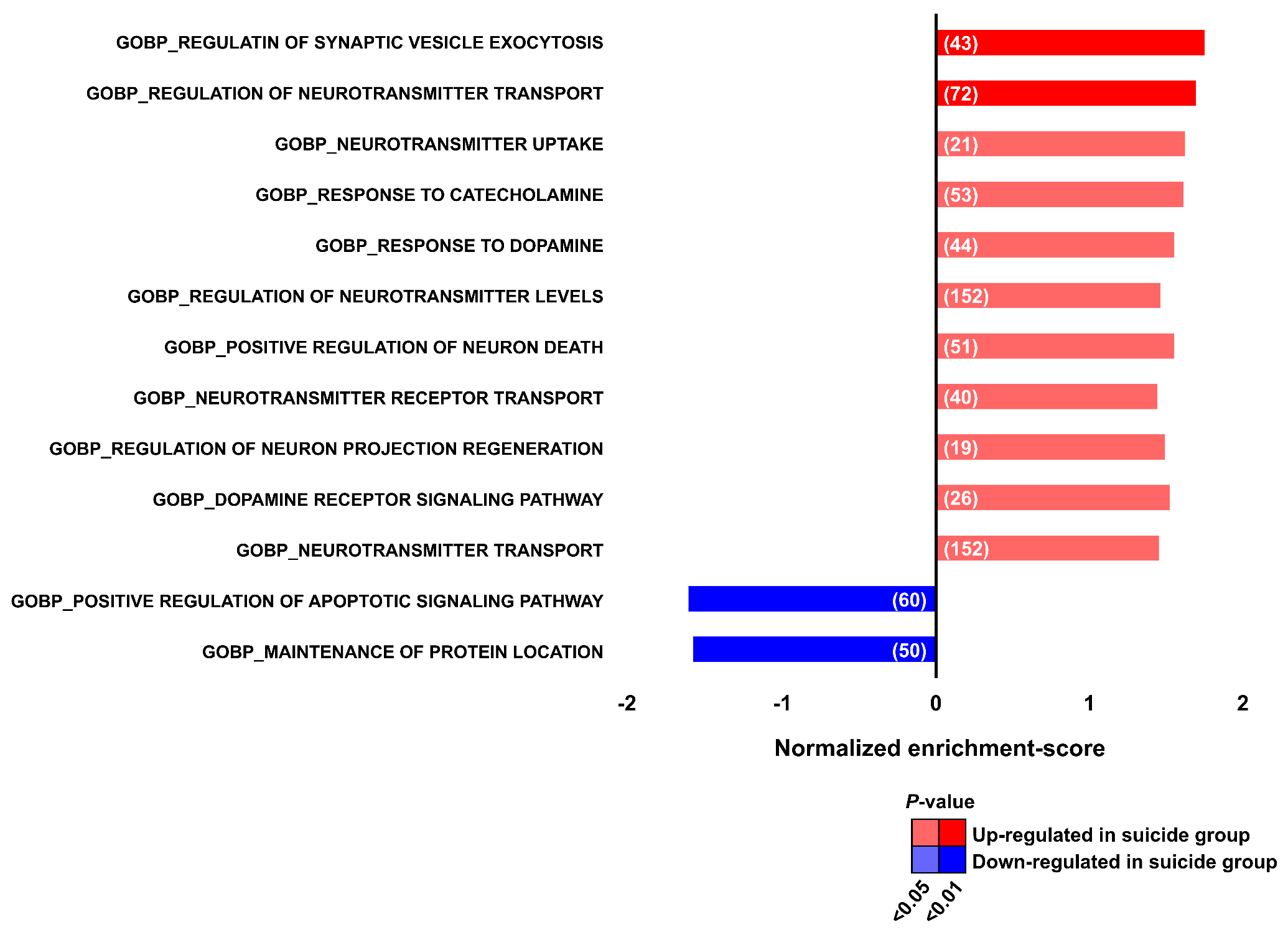


**Figure S5.** Gene set enrichment analysis (GSEA). GSEA linking differentially expressed genes to with the canonical pathway or biological process. Normalized enrichment scores (NES) of process are depicted.


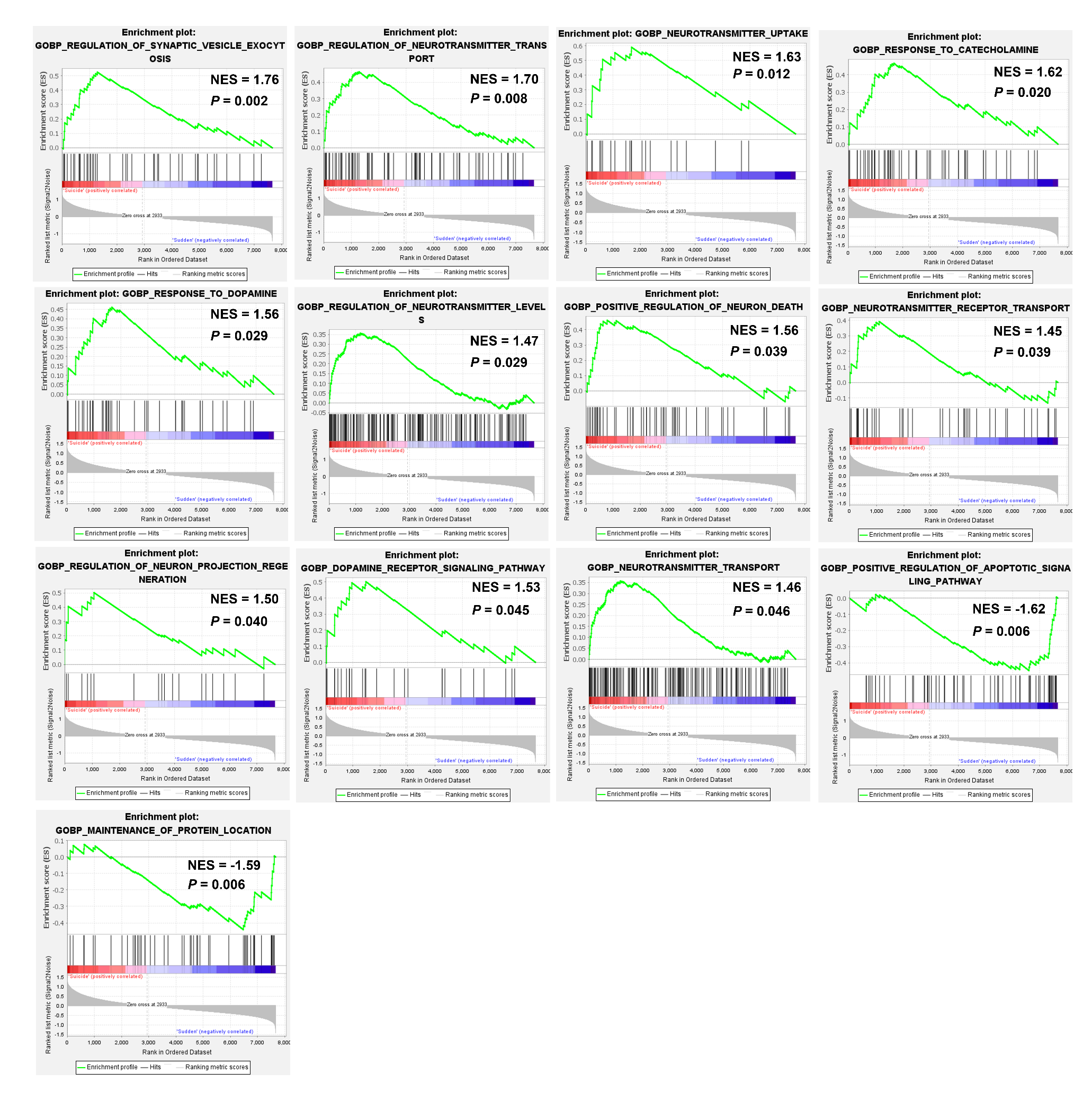


**Figure S6.** Gene set enrichment analysis (GSEA). GSEA Enrichment score curves are shown in green, which is the running sum of the weighted enrichment score obtained from GSEA software, while the normalized enrichment score (NES) and the corresponding P-value are reported within each graph.


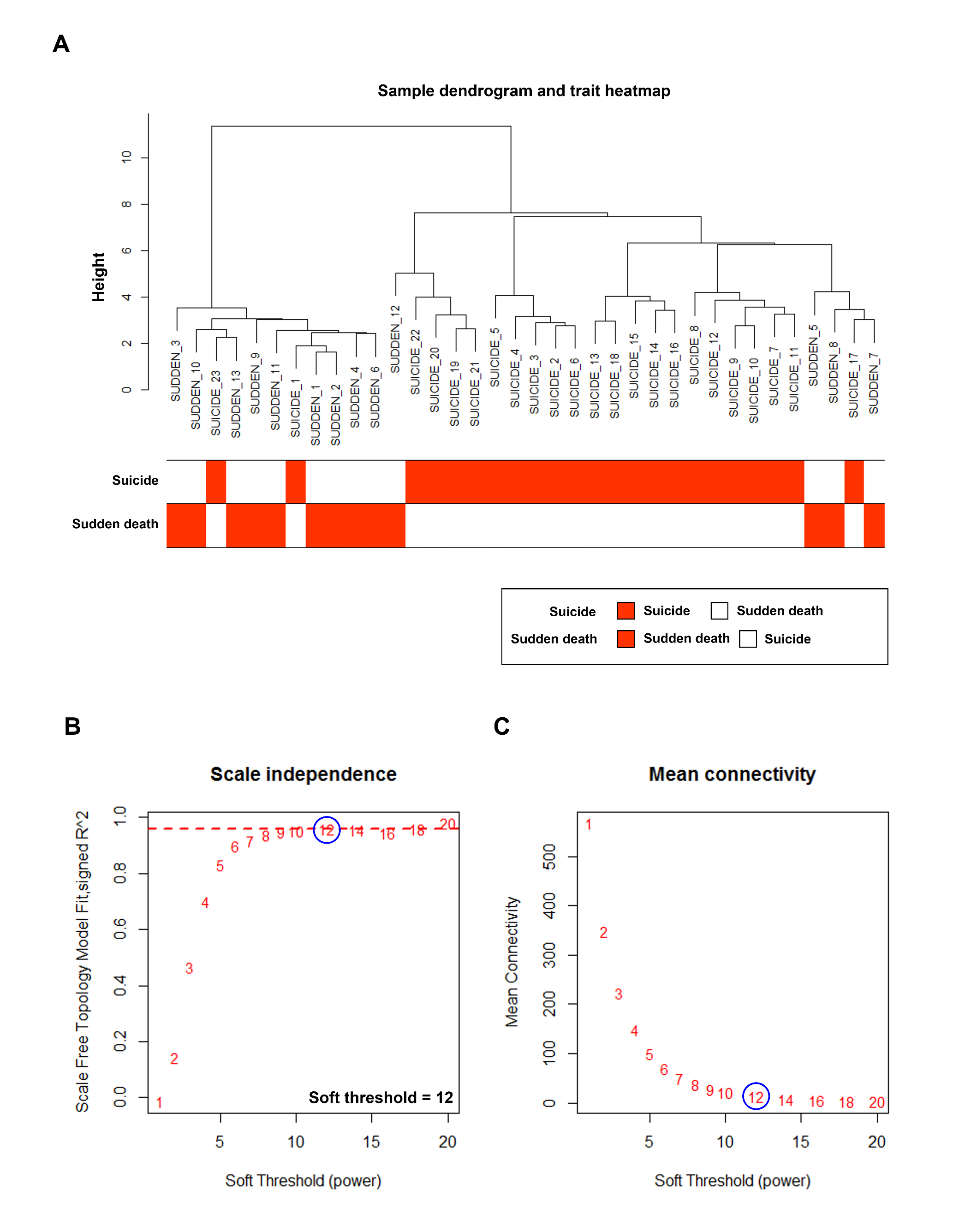
**Figure S7.** WGCNA analysis. A. Hierarchical clustering dendrogram of all samples B. Scale independence. C. Mean connectivity.


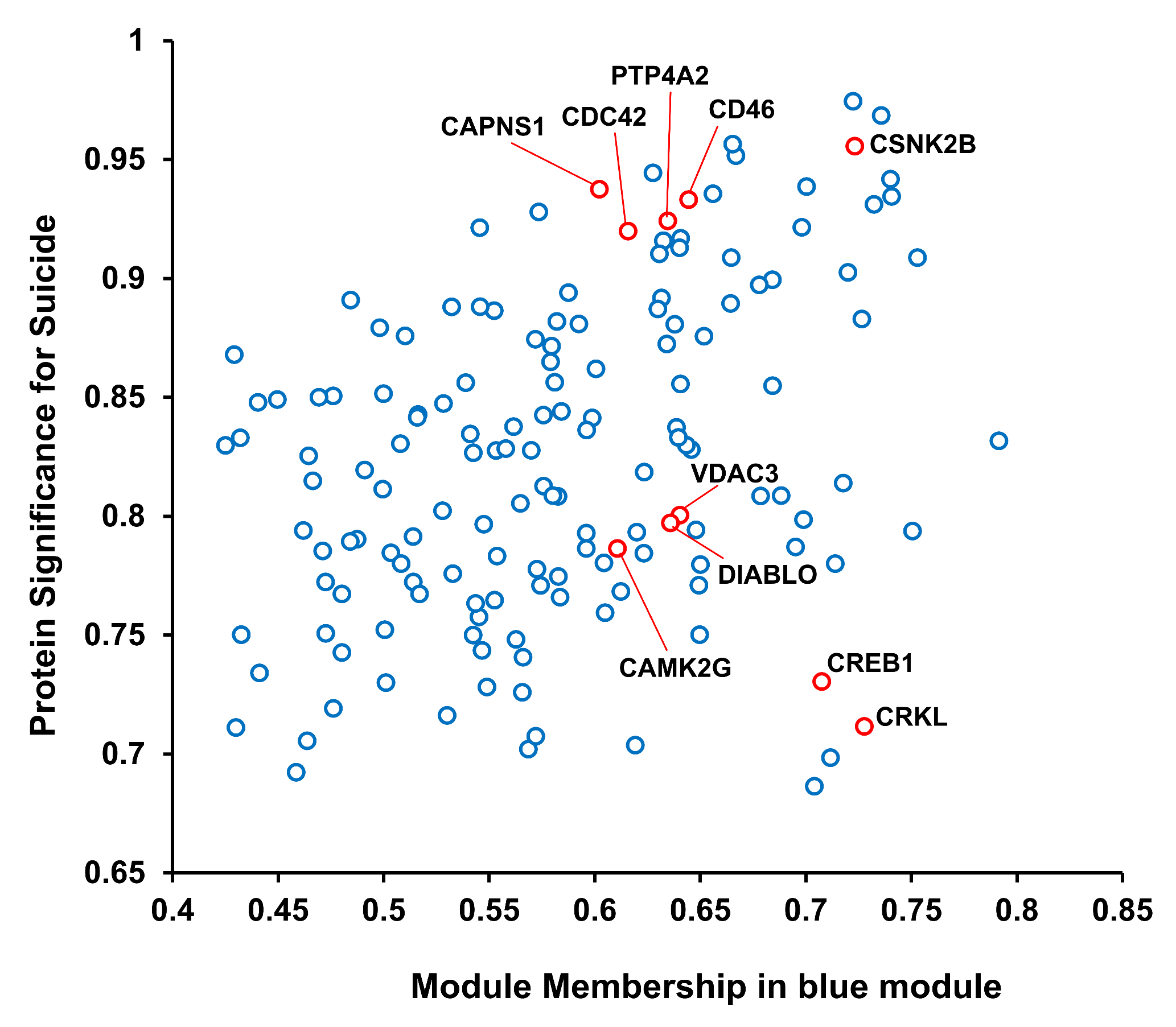


**Figure S8.** A scatterplot of protein significance for suicide versus module membership in the blue module.


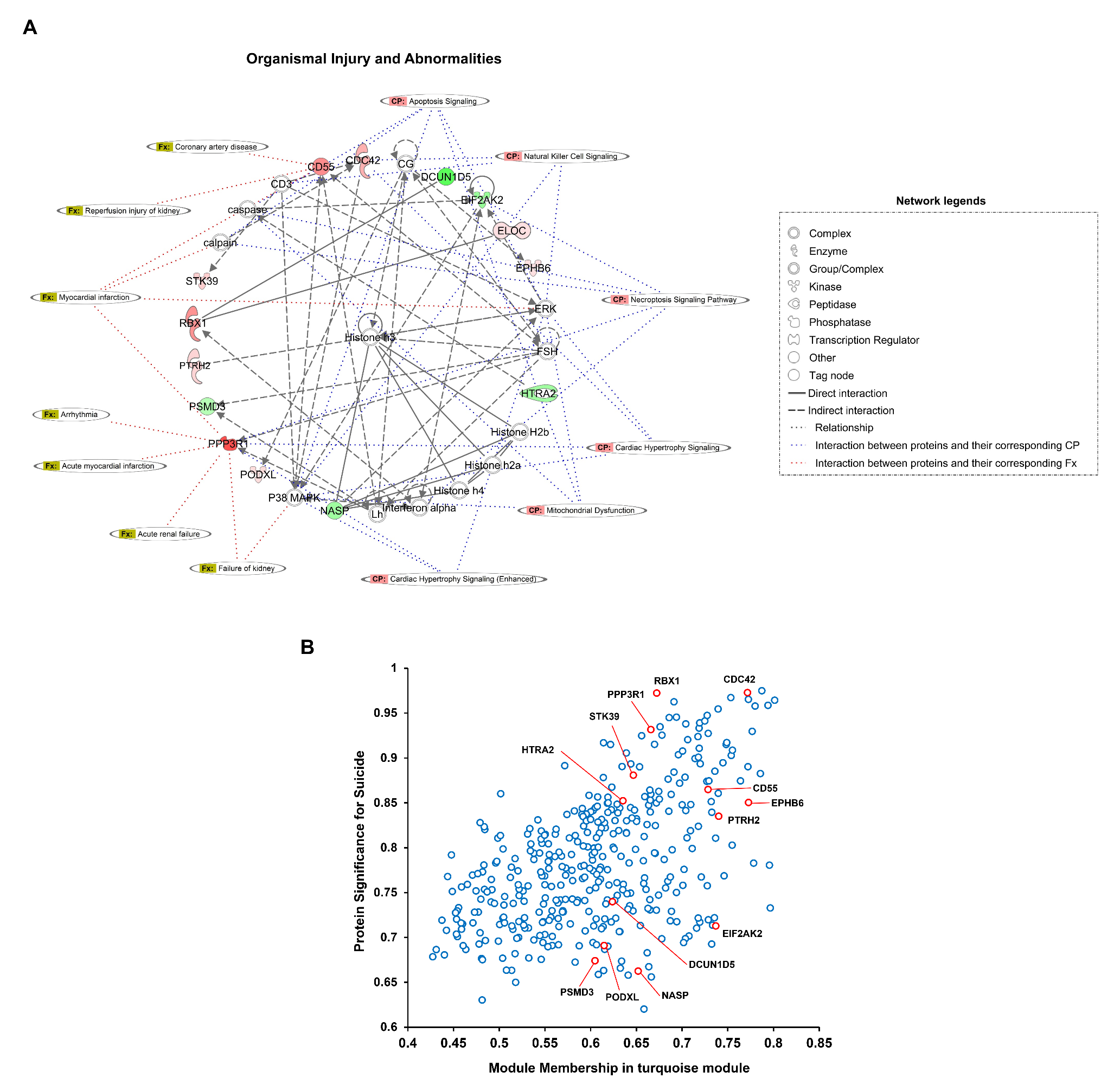


**Figure S9.** A. The top network using 393 proteins in the turquoise module that has the largest correlation with the sudden death group. Organismal injury and abnormalities pathway was identified B. A scatterplot of protein significance for suicide versus module membership in the turquoise module.


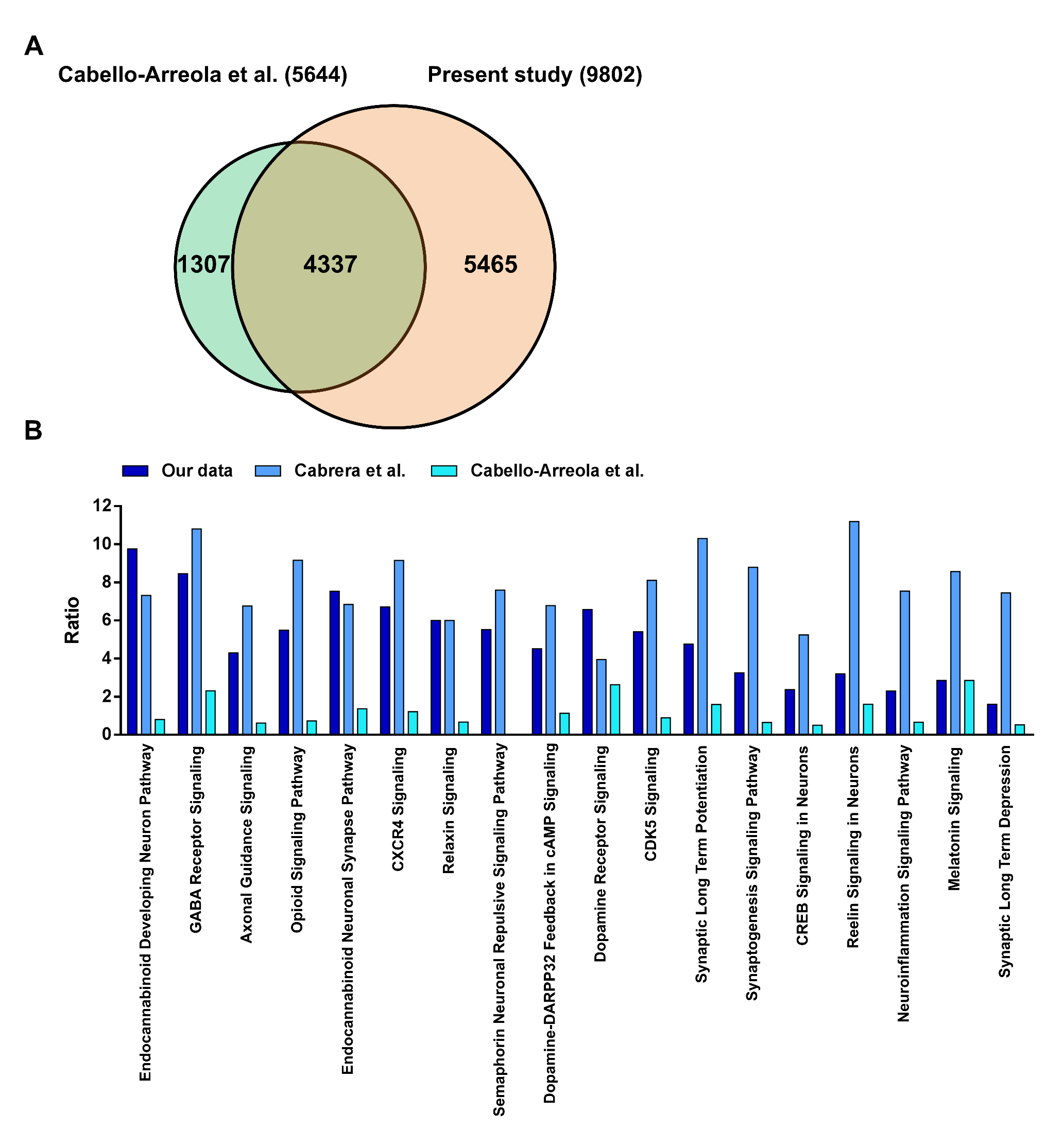


**Figure S10.** Comparative studies. A. Venn diagram to compare the protein profiles discovered in the previous study. B. Common IPA canonical pathways found in three studies. The ratio presented is defined as the number of the differentially expressed proteins or differentially expressed genes found in three studies over the total number of proteins involved in each of the pathway.

**
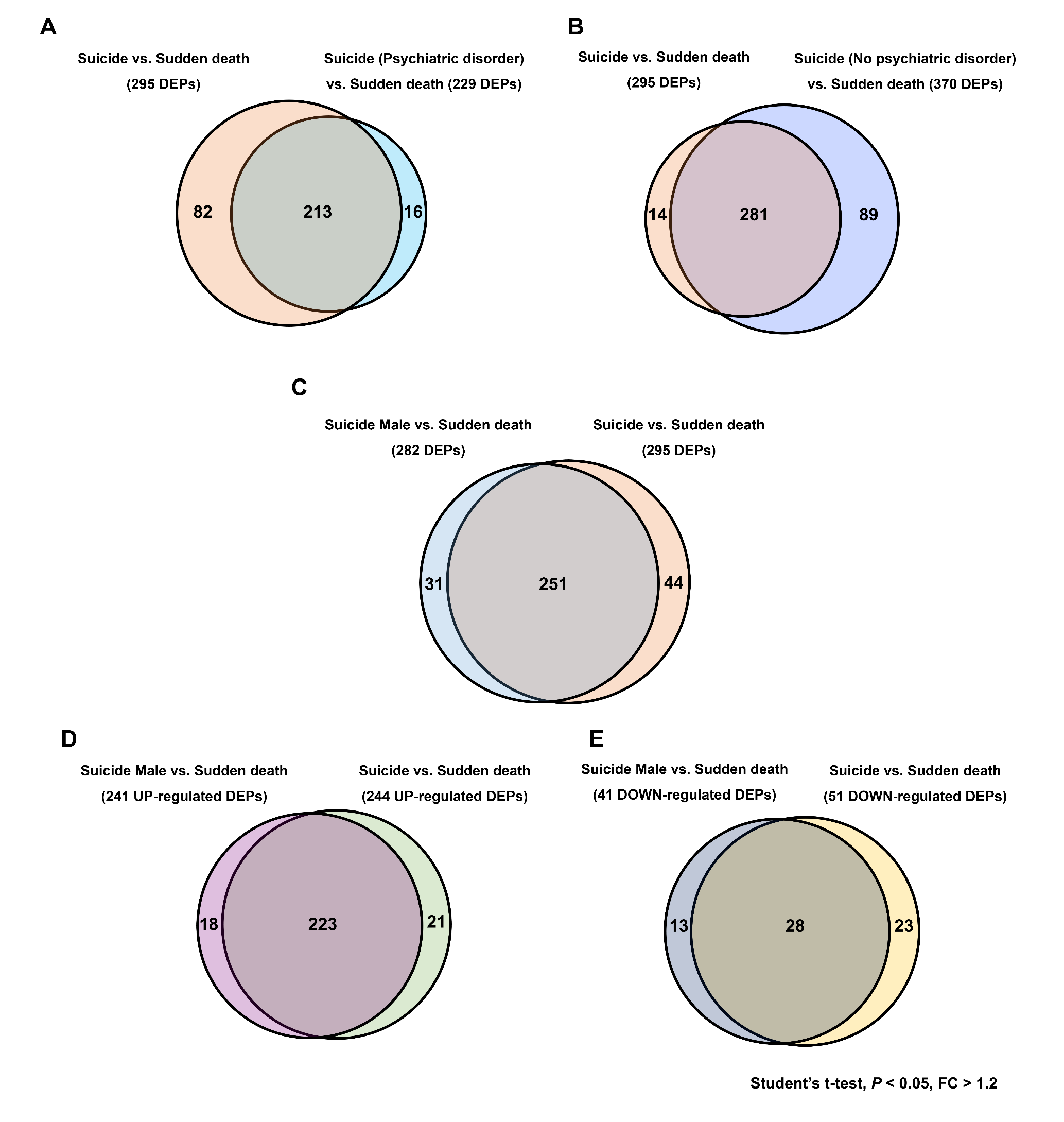
**

**Figure S11**. Venn diagrams for differentially expressed proteins between suicide (psychiatric disorders) vs. sudden death (A), and between suicide (no psychiatric disorders) vs. sudden death (B). Venn diagrams for differentially expressed proteins between suicide(all) vs. sudden death and suicide (male) vs sudden death group using all DEPs (C), up-regulated DEPs (D), and down-regulated DEPs (E).
